# Quantifying Human Visible Color Variation from High Definition Digital Images of Orb Web Spiders

**DOI:** 10.1371/journal.pone.0166371

**Published:** 2016-11-30

**Authors:** Horacio Tapia-McClung, Helena Ajuria Ibarra, Dinesh Rao

**Affiliations:** 1 Laboratorio Nacional de Informática Avanzada, A.C., Xalapa, Veracruz, México; 2 INBIOTECA, Universidad Veracruzana, Xalapa, Veracruz, México; City University London, UNITED KINGDOM

## Abstract

Digital processing and analysis of high resolution images of 30 individuals of the orb web spider *Verrucosa arenata* were performed to extract and quantify human visible colors present on the dorsal abdomen of this species. Color extraction was performed with minimal user intervention using an unsupervised algorithm to determine groups of colors on each individual spider, which was then analyzed in order to quantify and classify the colors obtained, both spatially and using energy and entropy measures of the digital images. Analysis shows that the colors cover a small region of the visible spectrum, are not spatially homogeneously distributed over the patterns and from an entropic point of view, colors that cover a smaller region on the whole pattern carry more information than colors covering a larger region. This study demonstrates the use of processing tools to create automatic systems to extract valuable information from digital images that are precise, efficient and helpful for the understanding of the underlying biology.

## Introduction

Digital images are created when light is captured using digital sensors that respond to the emitted and reflected photons of the various sources that make a scene. High resolution digital images record large amounts of information of the colors close to how they are perceived by the human eye and sometimes in other wavelengths, and have been used in biological studies of color patterns and variations among different individuals of the same species to help understand the relation between coloration and behavior [1–7]. Quantifying color as perceived by the human eye can help understand some features regarding the function of coloration on a particular species; for example, the study of spatial patterns in the distribution of colors allows for testing for discrete color morphology among individuals of a species [3, 6–11], for relations between morphology and the ecology [2–5, 8, 12, 13], aposematism, sexual selection, etc.

**Fig 1:**
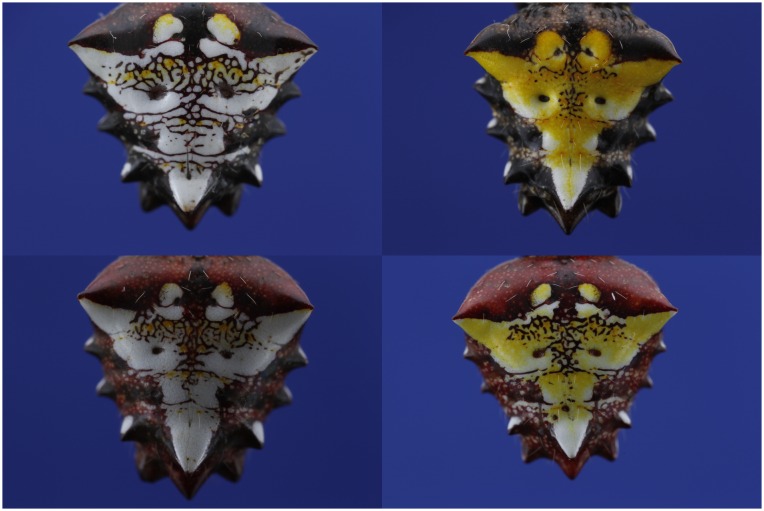
(Color online) A representative sample from the abdomens of 30 *Verrucosa arenata* individuals, captured using high resolution digital images. Patterns show a range of human visible color variation between extremes of white and yellow. Note, however, that other colors are present in the patterns.

Bright body coloration in orb web spiders has been thought to function as an insect lure [14]: pollinating insects are deceived by the flower-like patterns of the spiders and thus fall into the web. However, the same properties that make the spider attractive to prey, including bright color and reflection in the UV part of the spectrum also attract potential predators such as wasps and insectivorous birds, resulting in a trade-off between prey attraction and predator attention [15]. One of the fundamental characteristics necessary for successful deception is that the body patterns need not mimic a particular flower, but rather the generalized appearance of a flower. For example, in the case of the orchid mantis, flower seeking prey are significantly more likely to approach an individual even though its appearance does not mimic a particular orchid, but rather the general characteristics of an orchid [16]. In the case of orb web spiders, bright body coloration has been shown to attract insects in several spider species [14]. However, flowers are rarely colored uniformly. They consist of a series of variations in both color and distribution. For there to be successful deception and, thereby, an efficient visual lure, the spiders should not only mimic the common floral colors but also the color patterns. A first step towards modelling the appearance of the spider seen from the perspective of potential prey or predators requires the quantification of color patterns and their relative distribution on the body. This is possible using high resolution digital images. For this study, we used the species *Verrucosa arenata*, a color polymorphic orb web spider widely distributed across North America. A previous study [12] with this species showed that the two morphs, white and yellow (Fig 1), are sympatric and occur in a ratio of roughly 3:1 (white:yellow) in the population. White morphs are generally in better body condition than the yellow morphs but attract fewer prey. However, in that study, only the dominant color from a human perspective was used to categorize the two morphs, while in reality morphs can be placed on a continuum between the extremes of completely white and yellow. Furthermore, as can be seen in Fig 1, there are other colors present on the abdomen such as brown and black, making the categorization based on the human perception of dominant color subjective and insufficient to capture possible functions of the color pattern on the spiders.

In some studies of the distribution of colors in biological systems [17], the colors of interest are obtained by segmenting the digital images using threshold values selected according to the perceived presence or absence of a particular color. This approach has the disadvantage that the color is chosen subjectively, according to the human visual system. The high complexity and variability in composition of color signals is best suited for an unsupervised algorithm that segments labeled pixels according to the color represented by similar pixel values from a digital image, preventing a bias by selecting preset threshold values [17]. Usupervised algorithms are commonly used for digital image processing [18–20]. While the comprenhensive toolbox for image calibration and analysis described in [21] provides a set of techniques for digital image processing, it is best suited to perform specialized measurements towards estimating colors in non-human visual systems, including tools to linearize digital images and to map signal to animal cone-catch values, ultimately allowing for more objective measurements from digital images that are independent of the human visual system. The approach described here aims to complement the toolboox in the analysis of color pattern variation.

To provide new understanding of the presence of color variations among individuals of a species, and to help in classifying individuals using color features [12, 17, 22], in this work an unsupervised clustering algorithm is applied to high resolution digital images of individuals of the orb spider *Verrucosa arenata* to extract human visible colors present in the bright colour pattern on the abdomen of the spiders [12, 23].

After extracting the color information contained in the pixel values, measurements of the energy and the entropy of the digital image are obtained. Both image measurements have been used in different applications of image processing to evaluate the quality of digital images [24], and chemical properties extracted from digital images [20], among others. The energy quantifies the average intensity of the pixels and the entropy quantifies the uncertainty of information provided by the image; when entropy of a digital image increases, the information associated to the image is closer to random, when it decreases, it is less random [18–20].

Finally, the available pixel information is used to develop a basic model to detect regions with large color coverage in the patterns. The model is based on the probability that a region in the pattern contains pixels with color values corresponding to the color that is more prominent in the whole data set, thus an *a priori* probability can be assigned to that pixel; a probability distribution is estimated from the complete pixel data.

The remainder of the manuscript is divided into three main sections. The first section describes the methods for acquiring and processing the digital images. Next, the results of applying the unsupervised algorithm and the analysis of the extracted human visible color features of the patterns is presented, concluding with a discussion and plans for future work.

## Methods

A brief description of the image acquisition process is first presented, followed by the pre- and post-processing operations performed on the high resolution digital images, which are key to determining the regions containing the color patterns on the spiders and to extract the colors from the images. In this work we only look at human visible colors present in the patterns; whenever the word color is used throughout the text we refer to these frequencies. Colors are extracted using an unsupervised clustering method and processed to obtain energy and entropy measurements of each pattern. Finally, a basic probabilistic model to detect the color with the highest coverage in the patterns is developed from the image data.

### Image acquisition

A total of 30 adult females of the spider *Verrucosa arenata* (Araneae:Araneidae) [12, 23] were collected during December of 2014 at the Parque Ecológico El Haya in Xalapa, Veracruz, Mexico. Spiders were placed inside a cardboard box at a distance of 15 cm above the background, consisting of a blue chroma key fabric (Flashpoint^™^). Green, purple and blue backgrounds were tested, with the blue one providing the best contrast against the color pattern of the spiders. Digital images of the abdomen of each spider were acquired using a Canon EOS 7D camera fitted with an EF 100 mm f/2.8 USM macro lens, placed at a distance of 15 cm. from the spider.

A Macro Twin Lite MT—24 EX flash unit was used as illumination. Exposure settings were adjusted manually and kept constant for all images acquired. Photographs were recorded in both 16 -bit RAW and 8 -bit JPEG image device types. The RAW images where used for the analysis. Fig 1 shows four examples of the raw images. *V. arenata* is commonly known as the arrowhead spider, since the brightly colored part of the abdomen forms a distinct triangular shape. The spider varies in coloration not only in the pigmented part, but also in the surrounding area, see Fig 1. The abdomen is fringed by short spines of which two of the lower ones are white color irrespective of the morph.

### Image processing

Processing and analysis was performed using functions and tools available in the commercial software *Mathematica* [25] running on an 8M RAM Intel i7-3770 desktop computer with a 64-bit Linux Operating System. All the processing code and sample images are available on request.

The typical size of the 16-bit high resolution RAW images acquired is about 5194 pixels wide by 3457 pixels high. This level of resolution, about 18 million pixels per image, allows for a very detailed extraction of the colors present in the pattern captured by the digital image, but can also be a computationally expensive process. A pre-processing step performed to automatically determine a *region of interest* (roi) in the image consists of extracting the foreground containing the spider’s color pattern by removing the constant background. This process is efficient due to the high contrast between the colors in the patterns and the blue background used to acquire the digital images. Removal of the background from the original image leaves about 10 to 12 million pixels with information pertaining only to the color pattern on the spider abdomen. No image rescaling is necessary, thus avoiding data loss. The resulting images are processed, segmenting the image according to the values of each pixel using an unsupervised algorithm to determine groups of colors.

### Background removal and region of interest

**Fig 2:**
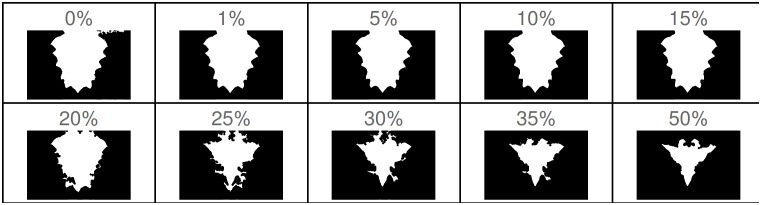
Effect of choosing different threshold values to remove the background. The threshold values shown represent a percentage of the average luminosity of the background (see main text). Values below a 10% of the mean luminosity show no significant difference in the extracted *region of interest* from the digital images.

As described previously, images were acquired using a high contrast chroma blue background, which is useful in determining the *region of interest*. A first approach to determine this region is to segment images using preset threshold values for the RGB triplet of the background. This approach can prune pixels from the region containing the color pattern if an inadequate threshold value is selected (see Fig 2).

**Fig 3:**
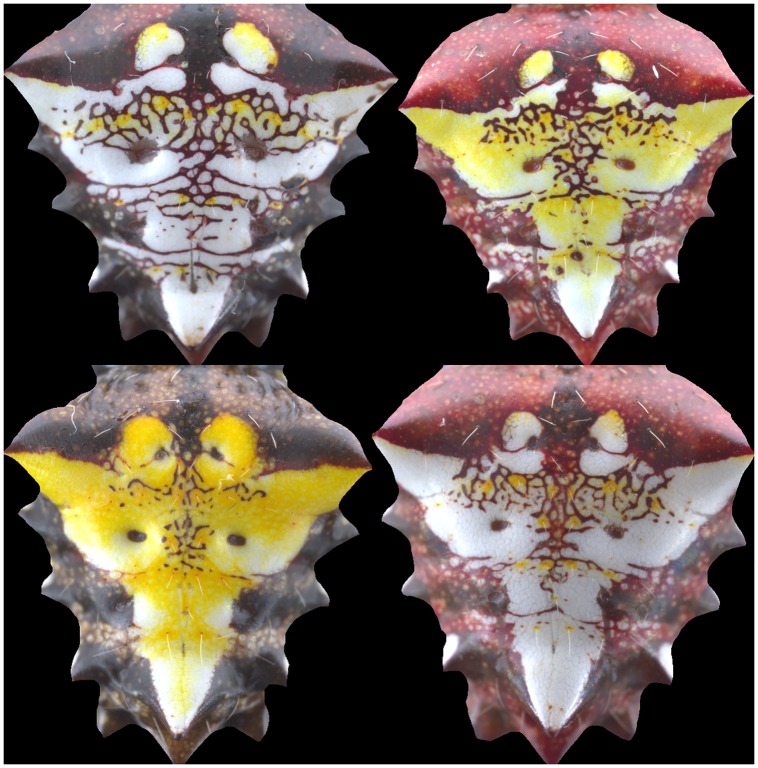
(Color online) Resulting *region of interest* containing the full color pattern obtained using the preprocessing steps described in the main text. These images are still very high resolution, about ≈ 60% of the original raw images (Fig 1).

A better approach is to select the threshold based on the mean luminance value of the background, obtained by averaging different pixels from regions with blue background in the image. In this work, for each image, pixels on vertical stripes along both edges of the original image are used to determine a threshold value based on the mean luminance. Choosing it in this manner makes the extraction of the pixels corresponding to the color pattern precise and efficient, taking into account possible light variations captured during the acquisition of the images. Fig 2 shows the effect of extracting the *region of interest* using different threshold values that are calculated as a percentage of the mean background luminance. In the figure, dark areas correspond to regions detected as background pixels, and white areas correspond to the region of interest. A large threshold value removes pixels that belong to the color pattern region. In general, a threshold value below 10% of the mean luminance of the background is enough to extract the foreground pixels containing the region of interest, and is the value used when processing all the original images in this work. The resulting *region of interest* for the example images of Fig 1 is shown in Fig 3.

After extracting the foreground containing the color patterns, the resulting images contain approximately 60% of the information of the original raw images before background removal, still leaving very high resolution images containing only the information on the colors that make the patterns.

### Color extraction

The *region of interest* containing the color patterns focuses on the abdomen of the spiders. This region of the body is included because the contrast between the color pattern and the abdomen may be biologically relevant.

The color extraction algorithm used to determine the colors on the digital images of each pattern is based on the *k-means* clustering algorithm. This method for grouping pixels partitions the image into clusters so that each pixel belongs to the cluster with the nearest mean [18, 19]. These clusters are obtained by measuring the distance between pixel values in a color space that separates gray-scale information from color information by entirely encoding the first in the values of a lightness variable, denoted by L*, and the latter in values of red minus green color and green minus blue colors, respectively denoted by a* and b*. This color space is know as the *L* * *a* * b* system [18]. In contrast to other color spaces like the HSI which also decouples the intensity (I) from the color information (Hue and Saturation), the *L* * *a* * b* color space was designed so the Euclidean distance corresponds with perceived differences between colors. The space is defined so that the colors are independent of the device on which they are displayed.

When applied to each image in the dataset, ten groups of pixels were determined that have color pixel values close to a centroid color (the mean). Following the idea of the *spatial granularity* analysis used by some authors to quantify the contribution of marking sizes to a given pattern in biological entities [9, 26], this color decomposition is referred to as the *color granularity* of the pattern.

**Fig 4:**
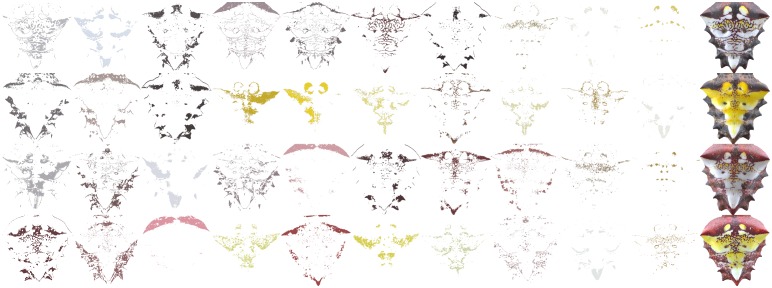
(Color online) Representation of the color granularity for the patterns of Fig 3.

The color granularity quantifies the amount of color and its spatial location on the abdomen of the spider ordered by area coverage on the pattern. Fig 4 shows the color granularity for the patterns of Fig 3. The color granularity contains information that provides a set of features of each pattern that can be used to characterize it instead of the ≈12 million pixel color values on each image.

## Probability model for color detection

A probability based model for the detection of the color with the largest covering in the pattern can be constructed from the color granularity. The model is built based on the *prior* probability for the color value of a pixel in the pattern. In this work, a very basic model is presented in which each pixel is classified either as belonging to the class with the highest coverage, or not.

To describe the model, *ω*_1_ denotes the state of the element of the color granularity with the highest coverage in the pattern, and *ω*_2_ denotes the state of any other element of the color granularity (any other with a lower coverage value). The *a priori* probabilities of a pixel being in either state *ω*_1_ or *ω*_2_ satisfy the rule *P*(*ω*_1_) + *P*(*ω*_2_) = 1 (uppercase *P* is used to denote *probabilities*, while lowercase *p* denotes *probability density functions*).

Consider the measurement *x* of some property of a pixel on the pattern; for example *x* could be the RGB color values. Taking these measured values to be a continuous random variable, the conditional probability density of *x* given that the state is the color granularity with the highest coverage, is described by a probability density function, *pdf*, expressed as *p*(*x*|*ω*_1_). Similarly the *pdf* of *x*, given that the state is any other color granularity, is denoted by *p*(*x*|*ω*_2_). Both probability density functions describe the difference in the property *x* between colors with the highest coverage and any other in the pattern. From Bayes’ formula, the probability for the element of the color granularity with the highest coverage in the pattern, *ω*_1_, given that the feature value *x* has been measured is:

$$\begin{array}{c} P\left(\omega_{1}|x\right)=\frac{p\left(x|\omega_{1}\right)P\left(\omega_{1}\right)}{p(x)} \end{array}$$ (1)

where the denominator

$$p\left(x\right)=p\left(x|\omega_{1}\right)P\left(\omega_{1}\right)+p\left(x|\omega_{2}\right)P\left(\omega_{2}\right)$$ (2)

is a normalizing factor that ensures that the probabilities add to 1. The *pdf* *p*(*x*|*ω*_1_) in the numerator of Eq 1 represents the *likelihood* that the element of highest coverage in the pattern *ω*_1_ is measured given that, or conditioned to, the property *x*’s value. Similarly, *p*(*x*|*ω*_2_) is the likelihood that any other element of the color granularity is measured, conditioned to the property *x*’s value. If *P*(*ω*_1_|*x*) > *P*(*ω*_2_|*x*) then the state *ω*_1_ is more likely to be the one to which the pixel with measured feature *x* belongs.

**Fig 5:**
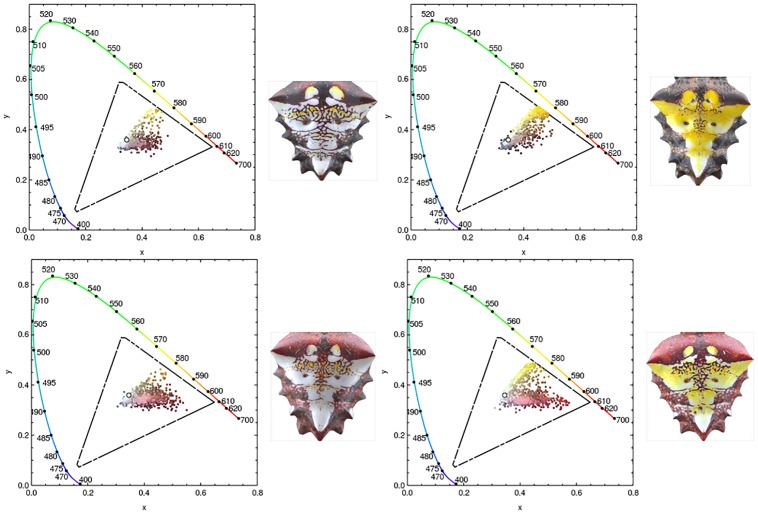
(Color online) Chromaticity plots showing the distribution of pixel color values corresponding to the patterns of Fig 1. The boundary is colored according to the visible spectrum; notice how the visible colors present in the patterns overlap covering only a small region of the *RGB* color space. The colors on the pattern cover a small portion of the RGB color space and they overlap, suggesting the use of a different color space to build the probability model.

**Fig 6:**
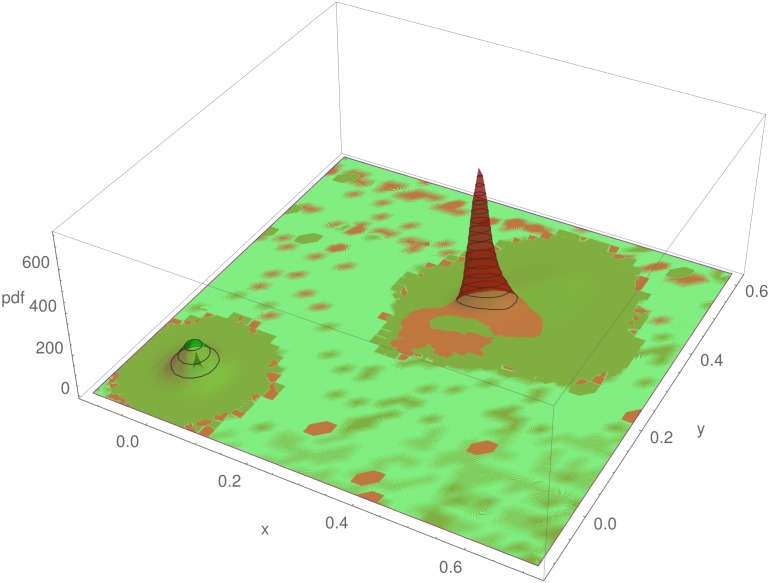
(Color online) Probability density functions*p*(*x*|*ω*_*j*_) for states of color with the highest coverage (*ω*_1_) of the pattern, and all the others (*ω*_2_), approximated by a two-dimensional distribution fitted to the chromaticity coordinates of the converted color data of the patterns.

Applying Eq 1 to the color values of each pixel in a pattern, the conditional probability density functions *p*(*x*|*ω*_1_) and *p*(*x*) can be approximated from the color granularity extracted from the pixel data of the 30 digital images as described in the previous section. The distribution of colors of the pixel values of each pattern covers a small portion of the RGB color space, as can be seen from the *RGB* color space *chromaticity diagrams* shown in Fig 5, where pairs of color coordinates inside a triangle in a two dimensional space containing all possible chromaticities in a given color space are shown [18, 19]. The boundary of the diagrams is displayed with the colors and wavelengths corresponding to the human visible spectrum. The diagrams show that the colors extracted from the pattern cover a small portion of the *RGB* color space and that they overlap, making it hard to differentiate the color that covers the largest portion of the pattern. To better distinguish colors on the pattern, the pixel values are transferred to the CIE XYZ color space, decomposing them into two chromaticity coordinates and one luminance coordinate. Keeping only the chromaticity coordinates, defined as

$$x=\frac{X}{X+Y+Z}$$ (3a)

$$y=\frac{Y}{X+Y+Z}$$ (3b)

the probability model expressed in Eq 1 is built in the two dimensional chromaticity plane color space defined by Eqs 3a and 3b. In order to train the statistical model to detect the colors with a larger coverage, the probability density functions *p*(*x*|*ω*_*j*_) are approximated by a two dimensional distribution fitted to the chromaticity coordinates given by Eqs 3a and 3b and shown in Fig 6 for the states of largest cover, *ω*_1_, and the others, *ω*_2_.

The *priors* *P*(*ω*_*j*_) are approximated as the total sum of pixels with the largest/others coverage for all the color granularity elements, divided by the total number of pixels in all the color granularity sets of the whole data corresponding to the 30 images.

The conditional probabilities *P*(*ω*_1_|*x*) and *P*(*ω*_2_|*x*) of test pixels are obtained and compared. If *P*(*ω*_1_|*x*) > *P*(*ω*_2_|*x*) then the testing pixel is classified as belonging to the element of the color granularity with the highest area coverage, otherwise, it does not belong to this element. In this way pixels in a pattern can be classified into pixels belonging to the highest coverage color granularity or not. The results of applying this simple probability-based pixel classifier are presented in the next section.

## Results

**Fig 7:**
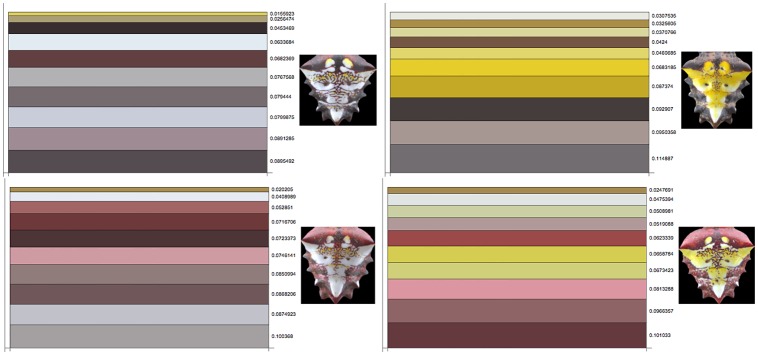
(Color online) Stacked bar charts showing the proportion of each color covering the *roi* for the sample patterns of Fig 3.

For each pattern in the database, the ten extracted colors are arranged and displayed in stacked bars in which the height of each bar is proportional to the percentage of coverage of that color in the corresponding pattern. The corresponding charts for the sample patterns of Fig 3 are shown in Fig 7. Each bar represents an element of the color granularity of a pattern. Each full stack is normalized to 1, meaning that all the colors displayed cover the whole region of interest; the numbers appearing at the side of each bar represent the percentage of coverage in the pattern of each color.

**Fig 8:**
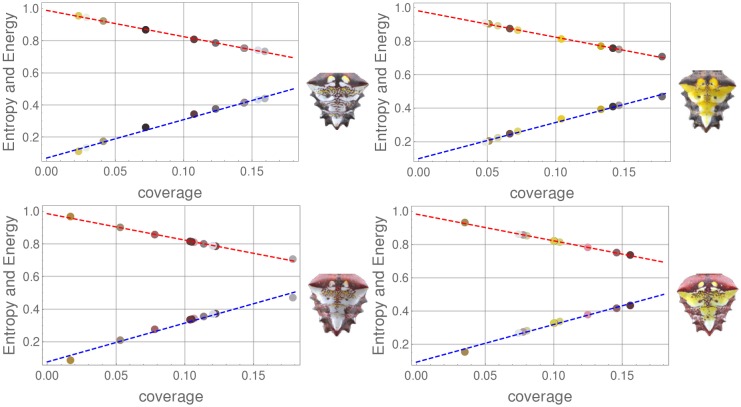
(Color online) Energy (top data points) and entropy (bottom data points) measurements and best line fit as a function of coverage area over the region of interest for the images of Fig 3.

The energy and the entropy associated to each color granularity element of a pattern are then measured. Fig 8 shows the values of these measurements on the color granularity of the sample patterns of Fig 3, plotted as a function of the coverage value of each element of the color granularity.

**Fig 9:**
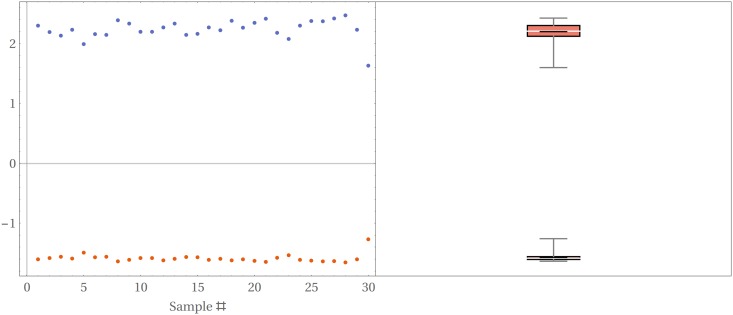
(Color online) Value of the slopes of the best fit lines to the energy/entropy data shown in Fig 8 for the whole image dataset.

Both energy and entropy vary monotonically with the coverage of the extracted color: energy/entropy decreases/increases for colors that cover larger areas over the patterns. This monotonic behavior for the measured values of energy/entropy is observed across all the patterns analyzed, as can be seen from the nearly constant values of the slopes of the best fit lines across the whole image dataset plotted in Fig 9.

**Fig 10:**
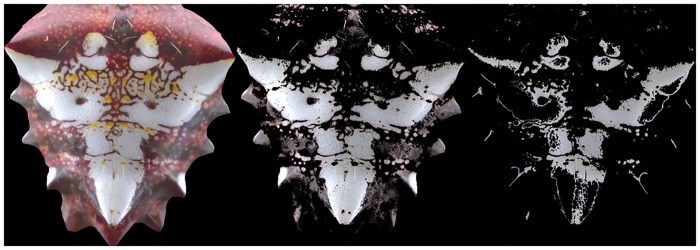
(Color online) Example showing the resulting region of highest coverage detected using the simple probabilistic method described in the main text. The left image is the original pattern, the middle image is the result of applying the probabilistic model and the image to the right shows the result of the unsupervised algorithm.

The basic probability model described above is trained using the whole data set of patterns. The *a priori* probability for the proportion of pixels corresponding to the largest coverage on the patterns was found to be *P*(*ω*_1_) ≈ .17 in the million—pixel training datasets. The *a priori* probability for all other colors in the data is *P*(*ω*_2_) = 1 − *P*(*ω*_1_) ≈ .83. The conditional probability distribution functions *p*(*ω*_*i*_|*x*) are approximated from the data by fitting a kernel distribution to the chromaticity coordinate pairs *x*, *y*, thus obtaining a function of the measured property *x*, which is applied to each pixel color value of the patterns in the database, resulting in the classified pixels shown in Fig 10.

The figure shows the result of applying the probability based method to detect the color with the largest coverage and a qualitative comparison to the result of the unsupervised clustering method for the same pattern. The probability method overestimates the region of highest color granularity in the pattern, but it is nevertheless promising that the corresponding color and region seems correctly detected, at least qualitatively. Because the probability model is very basic, precise results are not expected; acquiring and using more images corresponding to different individuals to train this model will provide better classification results in the future.

## Discussion

The method described proves to be an effective tool to automatically extract colors from patterns of the *Verrucosa arenata* spider. A key benefit compared to other approaches is its suitability for the automatization of color selection without human intervention, removing any subjectivity due to user perception of the color. The color quantification and analysis is carried out exclusively for human visible colors, as a first step in studying the patterns, before non-human visual systems like those of the spider’s predators are considered. Further work is required, but the approach used in this work could be incorporated into more comprehensive tools for performing detailed image analysis, such as that described in [21], which focus on other aspects of visual ecology and serve a different purpose. A difference from the approach described here is that there is no need to resize images and there is no requirement of uniformly scaled images for pattern analysis when comparing the patterns from different individuals.

The present study only looked at human visible colors. However, the analyses presented can be easily extended to false color images (that incorporate nonhuman visible wavelengths) generated by the toolbox described in [21]. Color information is of vital importance in many biological systems, and is used in processes such as sexual selection where the color of a male is actively evaluated by the female. Using an unsupervised algorithm on color patterns that are visible to the animal receiver can reveal focal points of selection pressures that have been hitherto ignored due to logistical difficulties.

Using the information about the extracted colors, the *color granularity* of the pattern is defined and used to quantify the amount and spatial location of colors of the spider’s abdomen. A simple probability-based model for detecting the largest covering color in the pattern is constructed and trained with available data, and then qualitatively compared with the results of the unsupervised method applied to extract the colors.

The color granularity of the patterns, Fig 4, and the stacked charts with the area coverage of each color, Fig 7, support the observation that brighter colors are usually the ones with lower coverage and are found inside the patterns, while colors with higher coverage are found in the body portion enclosing the brighter ones. Consequently, the appearance of the spider may serve to obscure its form and enhance the illusion [14, 15]. It is not yet clear if the area of the body enclosing the color region of the pattern serves some particular biological function. In flowers, the colors on the periphery play a distinct role in the process of attracting pollinators. Flowers with a bright centre and a dim periphery are potentially perceived as having blurred edges by honeybees [27]. The information on each color granularity set suggests that colors on the spider pattern are not distributed over a large range of wavelengths in the human visible spectrum, but rather span a small region of low wavelengths.

Energy and entropy measurements of all the color granularity sets are presented and show the same behavior across all patterns: the energy, a measure of the intensity of the pixels, monotonically decreases as the area covered by the color present on the pattern increases. The rate at which energy decreases with the increasing cover area appears to be the same for all patterns regardless of the particular color, remaining nearly constant with very small dispersion (Fig 8). Further work is required to understand if this is a feature of the color granularity as defined or an intrinsic feature of the patterns themselves.

Entropy, on the other side, increases monotonically with (increasing) coverage, a behavior observed for all the patterns analyzed. This feature, summarized in the plot showing the dispersion of the slope of the best line fits to the entropy on Fig 9, suggests an interpretation in terms of loss of information from the colors covering larger areas of the pattern, or alternatively, a gain of information from the color covering a smaller area. In probability terms, colors with lower coverage represent rare events and provide more information about the color pattern. A comparison of the perceived colors from the visual system of this species’ predator/prey can shed more light into the relevance of each color granularity and point towards the role of the colors with less coverage with respect to the biological functions of the pattern: if spiders mimic flower-like patterns, then the variations in color also matter.

Finally, regarding the probability model given by Eq 1, it is possible to generalize it to consider the conditional probabilities that the pixel to be tested belongs to any of the color granularity set, not just the one with the highest coverage. In this work the model was trained using only two conditionals: either it belongs to the color with highest coverage or not. The conditional probabilities can be better approximated using a larger data set thus improving the model. The basic model presented gives qualitatively acceptable results in classifying pixels as belonging to the region of highest coverage or not, and it shows that the data contained in the digital images are rich with information that can be analyzed using different approaches. Future work is underway to quantitatively test the model and refine it with more measurements and to continue combining morphological operations on digital images together with probability based methods using larger datasets to contribute to a better understanding of the color distributions on the spider’s pattern and their biological function.

## References

[1] Hyman J, Hansen M, Estrin D. Estimating the Spectral Reflectance of Natural Imagery Using Color Image Features. Workshop on Applications, Systems, and Algorithms for Image Sensing. 2008;.

[2] Kendal D, Hauser CE, Garrard GE, Jellinek S, Giljohann KM, Moore JL. Quantifying plant colour and colour difference as perceived by humans using digital images. PLoS ONE. 2013;8(8):e72296 10.1371/journal.pone.0072296 23977275 PMC3748102

[3] Taylor CH, Gilbert F, Reader T. Distance transform: a tool for the study of animal colour patterns. Methods in Ecology and Evolution. 2013;4(8):771–781. 10.1111/2041-210X.12063

[4] Garcia Mendoza J, Girard M, Kasumovic M, Petersen P, Wilksch P, Dyer A. Differentiating biological colours with few and many sensors: Spectral reconstruction with RGB and hyperspectral cameras. PLoS ONE. 2015;10(5):1–31. 10.1371/journal.pone.0125817 25965264 PMC4428825

[5] Garcia JE, Greentree AD, Shrestha M, Dorin A, Dyer AG. Flower colours through the lens: quantitative measurement with visible and ultraviolet digital photography. PLoS ONE. 2014;9(5):e96646 10.1371/journal.pone.0096646 24827828 PMC4020805

[6] Stevens M, PARraga CA, Cuthill IC, Partridge JC, Troscianko TS. Using digital photography to study animal coloration. Biological Journal of the Linnean Society. 2007;90(2):211–237. 10.1111/j.1095-8312.2007.00725.x

[7] Stevens M, Lown AE, Wood LE. Color change and camouflage in juvenile shore crabs *Carcinus maenas*. Frontiers in Ecology and Evolution. 2014;2:14 10.3389/fevo.2014.00014 PMC4281232 25551233

[8] Godfrey D, Lythgoe J, Rumball D. Zebra stripes and tiger stripes: the spatial frequency distribution of the pattern compared to that of the background is significant in display and crypsis. Biological Journal of the Linnean Society. 1987;32(4):427–433. 10.1111/j.1095-8312.1987.tb00442.x

[9] Chiao CC, Chubb C, Buresch K, Siemann L, Hanlon RT. The scaling effects of substrate texture on camouflage patterning in cuttlefish. Vision research. 2009;49(13):1647–1656. 10.1016/j.visres.2009.04.002 19362570

[10] Kemp DJ, Herberstein ME, Fleishman LJ, Endler JA, Bennett AT, Dyer AG, et al An integrative framework for the appraisal of coloration in nature. The American Naturalist. 2015;185(6):705–724. 10.1086/681021 25996857

[11] Marshall KL, Philpot KE, Damas-Moreira I, Stevens M. Intraspecific Colour Variation among Lizards in Distinct Island Environments Enhances Local Camouflage. PLoS ONE. 2015;10(9):e0135241 10.1371/journal.pone.0135241 26372454 PMC4570707

[12] Rao D, Castañeda-Barbosa E, Nuñez-Beverido N, Díaz-Fleischer F. Foraging benefits in a colour polymorphic neotropical orb web spider. Ethology. 2015;121(2):187–195. 10.1111/eth.12330

[13] Kang C, Stevens M, Moon Jy, Lee SI, Jablonski PG. Camouflage through behavior in moths: the role of background matching and disruptive coloration. Behavioral Ecology. 2015;26(1):45–54. 10.1093/beheco/aru150

[14] Tso IM. 23. In: Nentwig W, editor. Spider Ecophysiology. Upper Saddle River, NJ, USA: Springer-Verlag Berlin Heidelberg; 2013 p. 319–332.

[15] Fan CM, Yang EC, Tso IM. Hunting efficiency and predation risk shapes the color-associated foraging traits of a predator. Behavioral Ecology. 2009; p. arp064. 10.1093/beheco/arp064

[16] O’Hanlon J, Holwell G, Herberstein M. Predatory pollinator deception: Does the orchid mantis resemble a model species? Current Zoology. 2014;60(1):90–103. 10.1093/czoolo/60.1.90

[17] Teasdale L, Stevens M, Stuart-Fox D. Discrete colour polymorphism in the tawny dragon lizard (*Ctenophorus decresii*) and differences in signal conspicuousness among morphs. Journal of evolutionary biology. 2013;26(5):1035–1046. 10.1111/jeb.12115 23495663

[18] Gonzalez RC, Woods RE. Digital Image Processing (3rd Edition). Upper Saddle River, NJ, USA: Prentice-Hall, Inc.; 2006.

[19] Westland S, Ripamonti C. Computational colour science using MATLAB. John Wiley & Sons, Inc.; 2004 10.1002/0470020326

[20] Freire F, Kimura N, Luders D, Palangana A, Simões M. Calculation of the nematic entropy using digital images. Physical Review E. 2013;88(6):064502 10.1103/PhysRevE.88.064502 24483590

[21] Troscianko J, Stevens M. Image calibration and analysis toolbox–a free software suite for objectively measuring reflectance, colour and pattern. Methods in Ecology and Evolution. 2015;6(11):1320–1331. 10.1111/2041-210X.12439 27076902 PMC4791150

[22] Stevens M, Lown AE, Wood LE. Camouflage and individual variation in shore crabs (*Carcinus maenas*) from different habitats. PLoS ONE. 2014;9(12):e115586 10.1371/journal.pone.0115586 25551233 PMC4281232

[23] Rao D, Fernandez OC, Castañeda-Barbosa E, Díaz-Fleischer F. Reverse positional orientation in a neotropical orb-web spider, *Verrucosa arenata*. Naturwissenschaften. 2011;98(8):699–703. 10.1007/s00114-011-0811-2 21656002

[24] Tsai DY, Lee Y, Matsuyama E. Information entropy measure for evaluation of image quality. Journal of digital imaging. 2008;21(3):338–347. 10.1007/s10278-007-9044-5 17577596 PMC3043833

[25] Wolfram Research I. Mathematica version 10.3. Champaign, Illinois: Wolfram Research Inc.; 2015.

[26] Barbosa A, Mäthger LM, Buresch KC, Kelly J, Chubb C, Chiao CC, et al Cuttlefish camouflage: the effects of substrate contrast and size in evoking uniform, mottle or disruptive body patterns. Vision research. 2008;48(10):1242–1253. 10.1016/j.visres.2008.02.011 18395241

[27] De Ibarra NH, Vorobyev M. Flower patterns are adapted for detection by bees. Journal of Comparative Physiology A. 2009;195(3):319–323. 10.1007/s00359-009-0412-0 19184039

